# Reliability and Responsiveness of Clinical and Endoscopic Outcome Measures in Crohn’s Disease

**DOI:** 10.1093/ibd/izae089

**Published:** 2024-04-25

**Authors:** Reena Khanna, Brian G Feagan, Guangyong Zou, Larry W Stitt, John W D McDonald, Brian Bressler, Remo Panaccione, Lisa M Shackelton, Tanja VanViegen, Edward V Loftus, Marco Daperno, Vipul Jairath, Geert D’Haens, William J Sandborn

**Affiliations:** Department of Medicine, University of Western Ontario, London, Ontario, Canada; Department of Biostatistics and Epidemiology, University of Western Ontario, London, Ontario, Canada; Department of Medicine, University of Western Ontario, London, Ontario, Canada; Department of Biostatistics and Epidemiology, University of Western Ontario, London, Ontario, Canada; Alimentiv Inc., London, Ontario, Canada; Department of Biostatistics and Epidemiology, University of Western Ontario, London, Ontario, Canada; Alimentiv Inc., London, Ontario, Canada; Alimentiv Inc., London, Ontario, Canada; Alimentiv Inc., London, Ontario, Canada; Division of Gastroenterology, University of British Columbia, Vancouver, British Columbia, Canada; Department of Medicine, University of Calgary, Calgary, Alberta, Canada; Alimentiv Inc., London, Ontario, Canada; Alimentiv Inc., London, Ontario, Canada; Division of Gastroenterology and Hepatology, Mayo Clinic College of Medicine and Science, Rochester, MN, USA; Department of Internal Medicine (Division of Gastroenterology), Azienda Ospedaliera Ordine Mauriziano di Torino, Torino, Piemonte, Italy; Department of Medicine, University of Western Ontario, London, Ontario, Canada; Department of Biostatistics and Epidemiology, University of Western Ontario, London, Ontario, Canada; Alimentiv Inc., London, Ontario, Canada; Alimentiv Inc., London, Ontario, Canada; Department of Gastroenterology and Hepatology, Amsterdam University Medical Center, Location AMC, Amsterdam, The Netherlands; Division of Gastroenterology, University of California San Diego, La Jolla, CA, USA

**Keywords:** Crohn’s disease, outcome measures, operating properties

## Abstract

**Background:**

Regulatory guidance for Crohn’s disease trials recommends coprimary efficacy end points that evaluate both symptoms and mucosal inflammation. We aimed to characterize the operating properties of commonly used disease activity assessments alone and in combination.

**Methods:**

Endoscopic and clinical data were available for 129 participants from the Study of Biologic and Immunomodulator Naïve Patients in Crohn’s Disease trial. Readers scored the Simple Endoscopic Score for Crohn’s Disease and the Crohn’s Disease Endoscopic Index of Severity using standardized conventions. Index reliability was determined using intraclass correlation coefficients. Index responsiveness was assessed using standardized effect sizes based upon treatment assignment. Outcomes were evaluated for optimal sensitivity to treatment effect.

**Results:**

Substantial inter-rater reliability was observed when the Simple Endoscopic Score for Crohn’s Disease and Crohn’s Disease Endoscopic Index of Severity were used as continuous measures (intraclass correlation coefficient, 0.64; 95% confidence interval [CI], 0.50-0.73; and 0.62 95% CI, 0.36-0.77) compared with moderate reliability when dichotomized (0.46; 95% CI, 0.26-0.65; and 0.51; 95% CI, 0.00-0.78). The Simple Endoscopic Score for Crohn’s Disease, Crohn’s Disease Endoscopic Index of Severity, patient-reported outcome-2, and Crohn’s Disease Activity Index were similarly responsive (standardized effect size, 0.43, 95% CI, 0.05-0.81; 0.38, 95% CI, 0.0-0.76; 0.53, 95% CI, 0.15-0.91). A composite outcome of Crohn’s Disease Activity Index score <150 and Crohn’s Disease Endoscopic Index of Severity score <6 was most sensitive to treatment effect (28.9%; 95% CI, 11.0%-46.8%; *P* = .003).

**Conclusion:**

Endoscopic indices were more reliable as continuous measures. Composite outcomes including endoscopy improved sensitivity to treatment effect.

**Key Messages**

**What is already known?**

- Coprimary end points evaluating the signs and symptoms of Crohn’s disease (CD) and the underlying inflammatory disease process are currently mandated in clinical trials evaluating potential new therapies by regulatory agencies

**What is new here?**

- Endoscopic disease activity indices commonly used in CD clinical trials were most reliable as continuous measures and increased the sensitivity to treatment effect in composite outcomes that included clinical disease activity measures.

**How can this study help patient care?**

- These findings may assist in the design of efficient clinical trials and aid drug development for treatment of CD.

## Introduction

Coprimary end points that evaluate the efficacy of potential new therapies to improve both the signs and symptoms of Crohn’s disease (CD) and the underlying inflammatory disease process are currently mandated by both North American and European regulatory agencies.^1,2^ Consistency also generally exists between the US Food and Drug Administration (FDA) and the European Medicines Agency regarding the goals of therapy for this indication, namely clinical and endoscopic remission, both of which may be variably defined based upon the instrument/index used to measure disease activity. Furthermore, although remission is the ultimate endoscopic goal for treatment of CD, endoscopic response (also variably defined) is accepted as a component of the primary end point of CD clinical trials, given that not all therapies can achieve endoscopic remission within the time frame of a clinical trial, and there is a lack of data on the efficacy of currently approved therapies to achieve this outcome.

Several instruments to measure clinical and endoscopic disease activity have been developed and are employed to define both eligibility and end point criteria in randomized controlled registration trials. The most common of these include the Crohn’s Disease Activity Index (CDAI),^3^ the Simple Endoscopic Score for Crohn’s Disease (SES-CD),^4^ and the Crohn’s Disease Endoscopic Index of Severity (CDEIS).^5^ Although the CDAI includes valid symptom-based measures of clinical disease activity (abdominal pain [AP], stool frequency [SF]), and historic use of CDAI-based end points in phase 3 CD trials has led to regulatory approval for a number of therapies, concerns regarding the reproducibility and specificity of the CDAI have led to the use of interim measures of patient-reported outcomes (PROs), such as PRO2,^6^ until validated PRO measures for CD become available. Previous studies have confirmed the reliability and responsiveness of the SES-CD and the CDEIS, when used as continuous measures, identified common sources of disagreement among endoscopists and established conventions to minimize central reader variability.^7–9^ Nevertheless, several questions remain regarding the use of the SES-CD and CDEIS in clinical trials. For instance, the SES-CD and CDEIS are frequently dichotomized to categorize patients as having achieved, or not achieved, either clinical trial eligibility or outcomes of response and/or remission. Reliability estimates for these instruments when used as binary outcomes have not been reported.

A previous post hoc analysis^10^ of the Study of Biologic and Immunomodulator Naïve Patients in Crohn’s Disease (SONIC) trial^11^ suggested that a 50% improvement in the SES-CD score from baseline (endoscopic response) may have optimal sensitivity for defining a treatment effect; however, quantitative evaluation of this outcome, or other endoscopic outcomes defined based on the SED-CD or CDEIS when used in conjunction with clinical disease activity indices such as the CDAI or PRO2, has not previously been undertaken.^6^ Understanding the latter is critical for drug development for CD given current regulatory guidance. Data from the SONIC trial, which demonstrated superiority of infliximab (IFX) monotherapy and combination therapy with azathioprine (AZA) compared with AZA monotherapy, was used to evaluate these questions.

## Materials and Methods

### Study Population

Clinical data and endoscopic videos collected during the SONIC trial were used for the current study. In the SONIC trial, 508 patients with moderately to severely active CD naïve to immunosuppressive and biologic therapy were randomized to treatment with AZA (*n* = 170) or IFX (*n* = 169) monotherapy, or IFX and AZA combination therapy (*n* = 169). The primary outcome was corticosteroid-free remission at week 26, defined as a CDAI score of less than 150 points in patients who had not received ≥6 mg/day of budesonide or systemic corticosteroids for at least 3 weeks. A greater proportion of patients assigned to combination therapy achieved the primary outcome compared with those who received either IFX or AZA monotherapy (57%, 44%, and 30%, respectively, *P* = .02 for the comparison with IFX and *P* < .001 for the comparison with AZA). Mucosal healing (absence of ulceration larger than aphthous ulcers) at week 26 in patients with ulcers at the baseline colonoscopy also occurred more frequently in the combination therapy group compared with the IFX and AZA monotherapy groups (44%, 30%, and 17%, respectively); however, a significant treatment difference was only observed for the comparison of IFX and AZA combination therapy with AZA monotherapy (*P* < .001; *P* = .06 for the comparison with IFX monotherapy).

Data for the current study were generated from a substudy of the SONIC trial, in which 159 paired (baseline and week 26) video recordings of ileocolonoscopies marked by segment (rectum, descending and sigmoid colon, transverse colon, ascending colon, and terminal ileum), were collected from patients who had ulcers at baseline, as determined by a single central reader in the original trial. In the current study, 4 experienced gastroenterologists each screened approximately one-quarter of these videos to assess image quality. Of the 159 paired video recordings, 15 pairs were excluded due to poor video quality or inadequate bowel preparation, leaving 144 pairs available for evaluation. The presence of ulcers was confirmed by central reading based on the first read of a random reader (as is typical in the clinical trial setting) in 129 of 144 baseline videos. The final 129 video pairs formed the data set for the current study.

The study protocol was reviewed and approved by the Research Ethics Board at Western University. Informed consent obtained for the SONIC study included the use of this data for other medical purposes; therefore, additional patient consent for this study was not required.

### Study Objectives

The primary objectives of the study were to evaluate the operating properties of indices commonly employed in contemporary CD clinical trials and to determine the sensitivity of various endoscopic and clinical outcomes to treatment effect.

Specifically, we assessed the reliability of the SES-CD and CDEIS when used as continuous and binary measures, as well as the responsiveness of the SES-CD, CDEIS, CDAI, and PRO2. Sensitivity of clinical and endoscopic outcomes (when used independently or in combination) to treatment effect for AZA monotherapy relative to regimens including IFX (monotherapy and IFX and AZA combination therapy) was also assessed.

Secondary objectives included evaluation of methods for accounting for missing segments and the effect of excluding stenosis on the responsiveness of the SES-CD. Although calculation of a SES-CD total score is based upon assessment of index component items in all 5 ileocolonic segments, this is not always possible due to several reasons, including the presence of stricture, severe disease, previous bowel resection, or technical constraints. These factors may lead to variability in the number of segments assessed for individual patients at the baseline and follow-up ileocolonoscopies in clinical trials. Methods to adjust the SES-CD total score for the number of segments evaluated may minimize this source of variability, and their effects on index responsiveness were therefore also explored in this study. Finally, because stenosis is relatively infrequently encountered in patients included in clinical trials of moderately to severely active CD, is unlikely to respond to therapy, and has been associated with poor reliability,^7^ the effect of excluding this item on index responsiveness was also evaluated.

### Study Assessments

Signs and symptom-based measures of disease activity included the CDAI and PRO2.^6^ Endoscopic disease activity was assessed using the SES-CD and CDEIS.

Experienced central readers were trained on the use of the central image management system hosting the videos, as well as scoring of the SES-CD and CDEIS according to previously developed standardized conventions.^8,9^ For evaluation of index reliability, 4 central readers assessed 25 week-26 videos from the SONIC trial in triplicate. Videos used for this component of the study were presented in random order, and each assessment of the full set of 25 videos was separated by a minimum of 2 weeks. For evaluation of the responsiveness of the endoscopic indices, disease activity was evaluated in all paired baseline and week-26 videos in the absence of knowledge of treatment allocation, clinical information, or study visit.

The reliability of the SES-CD and CDEIS when used as continuous or binary measures was evaluated using scores calculated from standardized assessments of the SONIC videos by expert central readers.^7,9^ Responsiveness of the outcome measures (SES-CD, CDEIS, CDAI, PRO2) was quantified using effect sizes based upon treatment assignment, where assignment to either IFX monotherapy or IFX in combination with AZA was considered changed and assignment to AZA was considered unchanged. The sensitivity of multiple clinical, endoscopic, and composite outcomes to detect a treatment effect was evaluated and compared with the treatment effect observed for the original primary end point of the SONIC trial (corticosteroid-free remission [CDAI <150]) when data from all IFX-treated patients were pooled and compared with data from AZA-treated patients.

Evaluation of optimal methods for accounting for nonobserved intestinal segments included: 1) multiplying the mean of the available segment scores by 5; 2) imputation of missing values using the worst available segment score; 3) imputation of missing values using the maximum possible segmental score (12); 4) assessment limited to segments visualized at both baseline and week 26; and 5) adjustment for the number of segments observed in paired segments.

### Statistical Methods

Descriptive statistics were used to characterize the baseline demographics of the patients included in the study.

Reliability was assessed by calculating intraclass correlation coefficients (ICC), which are equivalent to weighted Kappa when data are ordinal. Point estimates for the ICCs were determined using a two-way random effects analysis of variance (ANOVA) model with interaction for the endoscopy video and reader, with both considered as random effects.^12^ Associated 95% confidence intervals (CIs) for the ICC estimates were obtained using the nonparametric percentile bootstrap method, commonly known as the cluster bootstrap method, with 2000 replicates sampled and replaced at the level of the video to maintain data structure. The degree of reliability was interpreted according to the benchmarks proposed by Landis and Koch whereby ICCs of < 0.00, 0.00–0.20, 0.21–0.40, 0.41–0.60, 0.61–0.80, above 0.80 indicate poor, slight, fair, moderate, substantial and almost perfect reliability.^13^

Responsiveness was quantified using Cohen’s effect size, defined as the difference in mean changes from baseline to week 26 between the two treatment groups divided by the pooled standard deviation (SD) of the changes. Benchmarks for small, medium, and large effect sizes are 0.2, 0.5, and 0.8, respectively.^14^ Index responsiveness was also estimated when the stenosis item was removed from the CDEIS and SES-SD, and when different approaches to account for nonobserved segments were utilized for scoring of the SES-CD.

The sensitivity of multiple dichotomous end points composed of endoscopic and clinical outcomes when used alone and as composite measures was assessed. The IFX-based treatment regimens evaluated in the SONIC study were assumed to be incrementally more effective than AZA.

## Results

### Study Population

Baseline demographic data for the patients whose video pairs were included in the analyses are summarized by treatment group in Table 1. The mean (SD) age of the patients was 35.5 (12.3) years, and 50.4% were male. Median disease duration was 2.2 years (interquartile range: 0.5, 8.5), and the mean (SD) CDAI score at baseline was 282.9 (50.9). A comparison of the baseline characteristics of the patients whose videos were included in the current study with those of the original SONIC patient population suggested that the 2 populations were similar, although disease duration was longer in patients treated with AZA monotherapy.

**Table 1.** Patient demographics and characteristics.

Characteristic	AZA Monotherapy (*n* = 39)	IFX Monotherapy (*n* = 45)	IFX and AZA Combination Therapy (n = 45)	All (*n* = 129)
Mean age, years (SD)	34.8 (12.3)	36.3 (14.0)	35.5 (10.7)	35.5 (12.3)
Male sex, *n* (%)	25 (64.1%)	19 (42.2%)	21 (46.7%)	65 (50.4%)
Caucasian, *n* (%)	33 (89.2%)	38 (92.7%)	36 (97.3%)	107 (93.0%)
Smoking status, *n* (%)				
Never smoked	15 (38.5%)	21 (46.7%)	18 (40.0%)	54 (41.9%)
Prior smoker	9 (23.1%)	5 (11.1%)	12 (26.7%)	26 (20.2%)
Current smoker	15 (38.5%)	19 (42.2%)	15 (33.3%)	49 (38.0%)
Disease duration				
Median years (Q1, Q3)	4.4 (0.6, 8.1)	1.8 (0.4, 5.6)	1.4 (0.6, 10.8)	2.2 (0.5, 8.5)
>3 years, *n* (%)	22 (56.4%)	16 (35.6%)	20 (44.4%)	58 (45.0%)
Disease location, *n* (%)				
Colon	10 (25.6%)	18 (40.9%)	10 (22.2%)	38 (29.7%)
Ileum	11 (28.2%)	9 (20.5%)	10 (22.2%)	30 (23.4%)
Ileum and Colon	18 (46.2%)	17 (38.6%)	25 (55.6%)	60 (46.9%)
Fistulas, *n* (%)				
Past	9 (23.1%)	8 (17.8%)	6 (13.3%)	23 (17.8%)
Present	7 (18.0%)	4 (8.9%)	5 (11.1%)	16 (12.4%)
Ever	13 (33.3%)	9 (20.0%)	10 (22.2%)	32 (24.8%)
Baseline corticosteroid ≥ 20 mg, *n* (%)	4 (10.3%)	7 (15.6%)	8 (17.8%)	19 (14.7%)
Mean CDAI (SD)	280.7 (50.0)	280.0 (48.2)	287.6 (54.9)	282.9 (50.9)
Median CRP concentration (Q1, Q3)	1.1 (0.3, 2.9)	1.9 (0.5, 3.5)	1.0 (0.5, 2.0)	1.2 (0.4, 2.9)

Abbreviations: AZA, azathioprine; IFX, infliximab; SD, standard deviation; Q, quartile; CDAI, Crohn’s Disease Activity Index; CRP, C-reactive protein.

### Operating Characteristics of the Endoscopic Indices

#### Reliability

The ICC estimates for intra- and inter-rater reliability of the SES-CD and the CDEIS based on assessment of week 26 videos were similar and consistent with substantial (ICC ≥0.61) intra- and inter-rater reliability (Table 2); ICC estimates (95% CI) for intra-rater reliability of the SES-CD and CDEIS were 0.80 (95% CI, 0.70-0.86) and 0.74 (95% CI, 0.59-0.84), and corresponding estimates for inter-rater reliability were 0.64 (95% CI, 0.50-0.73) and 0.62 (95% CI, 0.36-0.77). Removal of the stenosis item had no discernible effect on reliability estimates for either instrument. Importantly, numerical ICC estimates decreased for both instruments when they were used dichotomously, either as values typically employed to define clinical trial eligibility (SES-CD ≥7 and CDEIS ≥8) or those used as outcome criteria (SES-CD ≤4 or CDEIS <6) relative to those observed when either index was used as a continuous measure (Table 2).

**Table 2.** Reliability of the endoscopic indices.

	Intraclass Correlation Coefficients (95% CI)	
	Intra-rater Reliability	Inter-rater Reliability
**A. SES-CD**		
**Overall**		
Total score	0.80 (0.70, 0.86)	0.64 (0.50, 0.73)
Without stenosis	0.83 (0.75, 0.88)	0.69 (0.56, 0.77)
**Component items**		
Presence & size of ulcers	0.76 (0.62, 0.84)	0.60 (0.46, 0.71)
Extent of ulcerated surface	0.79 (0.67, 0.87)	0.73 (0.59, 0.83)
Extent of affected surface	0.74 (0.66, 0.81)	0.52 (0.29, 0.62)
Stenosis (presence/type)	0.63 (0.41, 0.74)	0.39 (0.22, 0.52)
**Eligibility and outcome criteria** ^a^		
SES-CD ≥ 7 (eligibility)	0.65 (0.51, 0.76)	0.43 (0.20, 0.60)
SES-CD ≤ 4 (outcome)	0.68 (0.53, 0.81)	0.46 (0.26, 0.65)
**B. CDEIS**		
**Overall**		
Total score	0.74 (0.59, 0.84)	0.62 (0.36, 0.77)
Without stenosis	0.77 (0.63, 0.86)	0.65 (0.41, 0.80)
**Component items**		
Deep ulceration	0.63 (0.37, 0.83)	0.35 (0.04, 0.67)
Superficial ulceration	0.71 (0.57, 0.79)	0.63 (0.44, 0.77)
Surface involved	0.71 (0.61, 0.81)	0.54 (0.28, 0.67)
Ulcerated surface	0.79 (0.74, 0.83)	0.68 (0.31, 0.76)
Stenosis	0.63 (0.34, 0.81)	0.51 (0.20, 0.72)
Any ulcerated	0.67 (0.10, 0.88)	0.54 (0.02, 0.82)
Any nonulcerated	0.47 (0.09, 0.69)	0.35 (0.04, 0.50)
**Eligibility and outcome criteria** ^a^		
CDEIS ≥8 (eligibility)	0.63 (0.00, 0.73)	0.48 (0.00, 0.59)
CDEIS <6 (outcome)	0.76 (0.37, 0.90)	0.51 (0.00, 0.78)

^a^ICC estimates reflect the reliability of the indices when used as binary outcomes to define classic thresholds used for trial eligibility and outcome criteria.

Abbreviations: CI, confidential interval; ICC, intraclass correlation coefficient; SES-CD, Simple Endoscopic Score for Crohn’s Disease; CDEIS, Crohn’s Disease Endoscopic Index of Severity.

The ICC estimates for the individual component items of both indices exceeded the threshold for substantial intra-rater reliability, except for any nonulcerated stenosis for the CDEIS, which was associated with moderate (0.47, 95% CI, 0.09-0.69) intra-rater reliability. The ICC estimates for the SES-CD component items were associated with at least moderate inter-rater reliability apart from scoring of stenosis, which was associated with fair inter-rater reliability (0.39; 95% CI, 0.22-0.52). The highest ICC estimate for inter-rater reliability of the SES-CD component items was observed for extent of ulcerated surface (0.73; 95% CI, 0.59-0.83). For the CDEIS, at least moderate inter-rater reliability was observed for the assessment of superficial ulceration, involved surface, ulcerated surface, and ulcerated stenosis, whereas fair inter-rater reliability was observed for the assessment of deep ulceration (0.35; 95% CI, 0.04-0.67) and any nonulcerated stenosis (0.35; 95% CI, 0.04-0.50). The highest ICC estimate for inter-rater reliability of the CDEIS component items was observed for ulcerated surface (0.68; 95% CI, 0.31-0.76).

#### Responsiveness

Standardized effect size estimates (95% CI) were similar for the SES-CD, CDEIS, CDAI, and PRO2 when change was defined based upon treatment assignment (0.43, 95% CI, 0.05-0.81; 0.43, 95% CI, 0.05-0.81; 0.53, 95% CI, 0.15-0.91; 0.38, 95% CI, 0.0-0.76, respectively; Table 3). The standard effect size for the SES-CD after exclusion of stenosis was relatively unchanged (0.40; 95% CI, 0.02-0.78) compared with the total score when stenosis was included. No consistent trend for standardized effect size estimates was observed when alternate methods were used to account for missing segments for scoring of the SES-CD (Table 4).

**Table 3.** Responsiveness of the indices to change at week 26 in patients with confirmed baseline ulcers (N = 129) based on treatment assignment^a^

Index	Standardized Effect Size (95% CI)
SES-CD	0.43 (0.05, 0.81)
CDEIS	0.43 (0.05, 0.81)
CDAI	0.53 (0.15, 0.91)
PRO2	0.38 (0.00, 0.76)

^a^AZA treatment group treated as reference or unchanged group; 90 patients changed; 39 patients unchanged.

Abbreviations: CI, confidential interval; SES-CD, Simple Endoscopic Score for Crohn’s Disease; CDEIS, Crohn’s Disease Endoscopic Index of Severity; CDAI, Crohn’s Disease Activity Index; PRO2, Patient-reported Outcome 2.

**Table 4.** Responsiveness of the SES-CD to change at week 26 in patients with confirmed baseline ulcers (N = 129) based on treatment assignment using alternative methods to account for missing segments

Method to Account for Missing Segment	Standardized Effect Size (95%CI)
Paired segments (adjusted for number of missing segments)	0.41 (0.03, 0.79)
Average of segments multiplied by 5	0.48 (0.09, 0.86)
Impute missing using worst available score	0.49 (0.11, 0.87)
Paired segments (total based on number of segments)	0.51 (0.13, 0.89)
Impute missing using maximum possible (12)	0.59 (0.20, 0.97)
Total score	0.43 (0.05, 0.81)

Abbreviations: CI, confidential interval; SES-CD, Simple Endoscopic Score for Crohn’s Disease.

### Sensitivity of Clinical and Endoscopic Outcomes to Treatment Effect

The sensitivity of various clinical, endoscopic, and composite outcomes to treatment effect with AZA compared with treatment including IFX (mono- and combination therapy) at week 26 in patients with ulcers at baseline of the SONIC trial is shown in Table 5. As expected, all outcomes evaluated favored treatment that included IFX, although not all were sensitive to detect a significant treatment difference compared with AZA. All clinical outcomes that included the CDAI detected a significant treatment difference between AZA and IFX; however, clinical remission, as defined by a CDAI score <150, was most sensitive to this effect (treatment difference, 28.3%; 95% CI, 10.1%-44.9%; *P* = .002). The primary outcome of the SONIC trial, the proportion of patients in corticosteroid-free clinical remission at week 26, was similarly sensitive (27.5%; 95% CI, 9.1%-44.1%; *P* = .003), whereas a decrease of ≥100 points in the CDAI score from baseline was least sensitive to treatment differences at week 26 (17.4%; 95% CI, 0.8%-34.6%; *P* = .040). Nonsignificant treatment differences were observed for all PRO2-based outcomes evaluated.

**Table 5.** Sensitivity of clinical and endoscopic outcomes to treatment effect.

Outcome	AZA (*n* = 39) *n* (%)	All IFX-treated (*n* = 90) n (%)	Difference % (95% CI)	*P* for difference
**Clinical**				
CDAI < 150	18 (46.2)	67 (74.4)	28.3 (10.1, 44.9)	.002
Corticosteroid free clinical remission (CDAI < 150)	17 (43.6)	64 (71.1)	27.5 (9.1, 44.1)	.003
CDAI decrease of ≥ 100	24 (61.5)	71 (78.9)	17.4 (0.8, 34.6)	.040
AP ≤ 1 and SF ≤ 3	21 (53.9)	58 (64.4)	10.6 (-7.3, 28.4)	.257
PRO2 < 8	22 (56.4)	60 (66.7)	10.3 (-7.3, 28.1)	.266
AP ≤ 1 and SF ≤ 1.5	16 (41.0)	45 (50.0)	9.0 (−9.6, 26.2)	.348
**Endoscopic**				
CDEIS < 6	21 (53.9)	73 (81.1)	27.3 (10.0, 44.0)	.001
SES-CD decrease > 50%	16 (41.0)	60 (66.7)	25.6 (7.0, 42.3)	.007
SES-CD ≤ 2	8 (20.5)	41 (45.6)	25.0 (7.1, 39.2)	.007
SES-CD ≤ 4 (≤ 2 for isolated ileal disease) AND no ulceration	15 (38.5)	54 (60.0)	21.5 (2.8, 38.1)	.024
SES-CD ≤ 4 (≤ 2 for isolated ileal disease)	17 (43.6)	57 (63.3)	19.7 (1.2, 36.8)	.037
SES-CD decrease > 25%	24 (61.5)	70 (77.8)	16.2 (-0.4, 33.5)	.057
**Composite (patient-level) clinical and endoscopic**				
CDAI < 150 AND CDEIS < 6	13 (33.3)	56 (62.2)	28.9 (11.0, 46.8)	.003
CDAI < 150 AND SES-CD ≤ 4	11 (28.2)	47 (52.2)	24.0 (6.5, 41.5)	.012
CDAI < 150 AND SES-CD decrease > 50%	12 (30.8)	49 (54.4)	23.7 (6.0, 41.4)	.013
CDAI < 150 AND SES-CD ≤ 2	6 (15.4)	34 (37.8)	22.4 (7.2, 37.5)	.012
AP ≤ 1 AND SF ≤ 3 AND SES-CD ≤ 2	5 (12.8)	29 (32.2)	19.4 (3.0, 31.9)	.022
PRO2 < 8 AND SES-CD ≤ 2	6 (15.4)	31 (34.4)	19.1 (2.1, 32.2)	.028
AP ≤ 1 AND SF ≤ 3 AND CDEIS < 6	13 (33.3)	47 (52.2)	18.9 (0.2, 35.1)	.048
PRO2 < 8 AND CDEIS < 6	14 (35.9)	49 (54.4)	18.6 (-0.2, 35.0)	.053
AP ≤ 1 AND SF ≤ 3 AND SES-CD ≤ 4	10 (25.6)	39 (43.3)	17.7 (-0.6, 32.8)	.057
PRO2 < 8 AND SES-CD ≤ 4	11 (28.2)	40 (44.4)	16.2 (-2.2, 31.8)	.083
PRO2 < 8 AND SES-CD decrease > 50%	12 (30.8)	43 (47.8)	17.0 (-1.0, 34.8)	.073

Abbreviations: AP, abdominal pain; AZA, azathioprine; CDAI, Crohn’s Disease Activity Index; CDEIS, Crohn’s Disease Endoscopic Index of Severity; CI, confidential interval; IFX, infliximab; PRO2, Patient-reported Outcome 2; SES-CD, Simple Endoscopic Score for Crohn’s Disease; SF, stool frequency.

Significant (*P* < .037) treatment differences at week 26 were observed for all SES-CD- and CDEIS-based endoscopic outcomes evaluated, except for a 25% decrease in the total SES-CD score from baseline (16.2%; 95% CI, −0.4% to 33.5%; *P* = .057). The endoscopic outcome most sensitive to a treatment effect was a CDEIS score <6 (27.3%; 95% CI, 10.0%-44.0%; *P* = .001). Nearly equivalent treatment differences were observed for a 50% decrease in the total SES-CD score from baseline and SES-CD ≤2 (25.6%; 95% CI, 7.0%-42.3%; and 25.0%, 95% CI, 7.1%-39.2% for the outcomes, respectively; *P* = .007 for both comparisons between treatment groups). However, fewer patients in both treatment groups achieved the latter outcome. Approximately half of the patients treated with AZA and two-thirds of those treated with IFX mono- and combination therapy met the outcome of endoscopic remission defined as a total SES-CD score ≤2 at week 26 relative to those who met the outcome of a 50% decrease in the total SES-CD score from baseline (16 [41.0%] and 60 [66.7%] vs 8 [20.5%] and 41 [45.65%] for AZA- and IFX-treated patients for the 2 outcomes, respectively).

The sensitivity of various composite (patient-level) clinical and endoscopic outcomes to detect a treatment effect at week 26 were also evaluated. The composite outcome most sensitive to detect differences between treatment with AZA and IFX at week 26 in patients with baseline ulcers comprised a CDAI score <150 and a CDEIS score <6 (28.9%; 95% CI, 11.0%-46.8%; *P* = .003), which was also the outcome most sensitive overall to treatment effect amongst all outcomes evaluated. Significant (*P* < .048) and similar (range, 18.9%-24.0%) treatment differences were observed for the composite outcomes of CDAI <150 and SES-CD ≤4, CDAI <150 and SES-CD decrease >50%, CDAI <150 and SES-CD ≤2, AP ≤1 *and* SF ≤3 and SES-CD ≤2, PRO2 <8 and SES-CD ≤2, and AP ≤1 *and* SF ≤3 and CDEIS <6. Although some composite outcomes including PRO2-based definitions detected significant treatment differences between AZA- and IFX-treated patients, they were generally less sensitive to treatment effect than composite outcomes that included CDAI-based definitions. For example, the treatment difference for the composite outcome of PRO2 <8 and CDEIS <6 was 10% lower (18.6%; 95% CI, −0.2% to 35%; *P* = .053) compared with the composite outcome of CDAI <150 and CDEIS <6.

## Discussion

The primary end point in CD registration trials has historically been a clinical or symptom-based outcome. However, it is now well established that CD symptoms are poorly correlated with active disease defined by objective measures such as endoscopy.^15^ The use of responsive measures of endoscopic disease activity has the potential to reduce the sample size required to detect treatment efficacy in clinical trials. Additionally, regulatory agencies have recommended the use of PROs to quantify the patient perspective/experience in conjunction with endoscopic measures of disease activity to evaluate the efficacy of new treatments. Few studies have evaluated the operating properties of the most commonly employed clinical and endoscopic indices when used in this manner. It has also previously been unknown whether the use of composite clinical and endoscopic outcomes on a patient level would obscure treatment effects, including those expected for highly effective agents such as IFX. In this regard, the SONIC data set was well-suited to evaluate these research questions. Based on the current study, 3 key messages have emerged.

First, ICC estimates for inter-rater reliability of the SES-CD and CDEIS decreased when these indices were employed as dichotomous vs continuous measures. This reduction in instrument precision is consistent with statistical principles that suggest continuous data are generally more robust. This finding is important because endoscopic scores are frequently dichotomized in phase 3 trials to determine eligibility and treatment efficacy. Our data thus provide important information for both regulatory agencies and pharmaceutical sponsors regarding optimal clinical trial design. Although ICC estimates for the reliability of the SES-CD and CDEIS were consistent with substantial reliability when they were assessed as continuous measures in the current study, these estimates were lower compared with previously published studies.^7,9^ Furthermore, during the extensive process of reevaluating these videos, it was apparent that the overall quality of the SONIC videos was substantially less than videos obtained in more contemporary studies. It is noteworthy that guidance to standardize bowel preparation, endoscopic washing, procedure cinematography, and recording of videos was lacking at the time the SONIC trial was undertaken.

Also of note, exclusion of stenosis from the endoscopic indices resulted in nonsignificant numerical improvements in ICC estimates but did not change the interpretation of the degree of reliability of the indices based on benchmarks defined by Landis and Koch.^13^ We had previously identified assessment of stenosis as contributing to lower ICC estimates for inter-rater reliability of the indices.^7^ Relatively poor reliability for the assessment of stenosis in endoscopy video recordings has largely been attributed to a lack of tactile feedback associated with passage of an endoscope through a narrowed area. Additional challenges for the assessment of stenosis in endoscopic video recordings include inconsistent ability to determine whether air or water insufflation has been attempted or if narrowed segments are optimally distended. Finally, the infrequent occurrence of stenosis in patients enrolled in CD clinical trials was a likely contributor to nonsignificant differences in ICC estimates observed with exclusion of this item from the indices. The relative infrequency of missing segments similarly contributed to our inability to define an optimal strategy to account for missing segments in the calculation of the SES-CD. These findings are consistent with the view that endoscopy is suboptimal for the evaluation of fibrostenosing disease and that consideration should be given to removing assessment of stenosis from future endoscopic indices of CD activity/severity. Our findings also underscore the value of cross-sectional imaging for this purpose,^16^ particularly as the evaluation of antifibrotic therapy for the treatment of CD gains momentum.

Second, the indices most commonly used to measure symptoms (CDAI, PRO2) and endoscopic disease activity (SES-CD, CDEIS) in clinical trials were similarly responsive to change defined by treatment assignment (AZA or IFX mono- and combination therapy). The CDAI demonstrated numerically greater effect sizes in this study compared with PRO2, and this observation was further reflected in our analysis of the sensitivity of various outcomes to treatment effect. However, recent phase 3 trials of upadacitinib and risankizumab for the treatment of CD did not show a similar consistent trend.^17,18^

Third, a clinical outcome of CDAI <150 and an endoscopic outcome of CDEIS <6 were individually most sensitive to treatment effect at week 26 amongst the clinical and endoscopic outcomes evaluated, and a composite of these outcomes on the patient level was the outcome most sensitive to treatment effect overall. However, the sensitivity of this pairing was not substantially greater than the homologous SES-CD-defined composite. These results suggest that these clinical and endoscopic outcomes, when used in combination to define clinical trial end points at the patient level, may increase the sensitivity of either outcome separately or when used as coprimary end points to detect treatment differences. The large treatment effect observed for a CDEIS score <6 may be explained, in part, by the use of continuous variables to measure affected and ulcerated surface area in this index in distinction to the use of categorical variables in the SES-CD, although the treatment effect observed for the most commonly used definition of response in contemporary clinical trials (a decrease of 50% in the SES-CD score from baseline) was highly similar and not significantly different from the CDEIS-based outcome. Additionally, calculation of the CDEIS is generally considered cumbersome, and a higher proportion of patients in both treatment groups achieved this outcome compared with endoscopic outcomes based on the SES-CD (with the exception of patients treated with AZA for the outcome of SES-CD decrease >25%, an outcome that has been previously shown to inadequately discriminate between active treatment and placebo^19^), which may reflect a reduced discriminatory capacity of the CDEIS relative to the SES-CD. Further exploration into potential reasons for this observation is required.

Importantly, most outcomes currently recommended by regulatory agencies for use as components of coprimary outcomes in CD clinical trials (CDAI <150, 50% decrease in the total SES-CD score from baseline, SES-CD ≤2, SES-CD ≤4) were sensitive to treatment effect at week 26 and detected significant differences between treatment with AZA and treatment including IFX. Our results also demonstrate substantially reduced sensitivity for outcomes based on the AP and SF components of the CDAI when used in isolation, as well as for the outcome of PRO2 <8, to detect significant treatment effects relative to clinical outcomes defined based on the CDAI. These observations, in theory, support current draft FDA guidance for the assessment of clinical remission (defined as CDAI <150) as a component of a coprimary end point in CD clinical trials and may suggest a benefit of the CDAI to increase sensitivity of both coprimary and composite end points to detect treatment differences in preference to PRO2. However, these observations may be specific for treatment including IFX, one of the most potent therapies available for the treatment of CD. Furthermore, these outcomes have not been substantiated in more recent trials. With regard to the endoscopic component of coprimary end points, a decrease in the SES-CD score of greater than 50% from baseline and an SES-CD score ≤2 were equally sensitive for the detection of a treatment effect at week 26; although fewer patients in both groups achieved the latter outcome. It is unknown whether use of a “true” placebo group would have further increased the sensitivity of the SES-CD ≤2 to detect a treatment effect; however, use of this outcome as an end point during the maintenance phase of CD clinical trials, as currently recommended by regulatory agencies, can currently be supported by our data. Finally, combined use of these endoscopic outcomes with CDAI-defined clinical remission (CDAI <150) was also sensitive to treatment effect at the patient level; treatment differences at week 26 were significant and highly similar when SES-CD decrease >50% or SES-CD ≤2 were included as composite outcomes with CDAI <150.

This study has some limitations. As alluded to previously, these results are based upon data from a single trial that did not include a placebo treatment for comparison. However, the SONIC study was a rigorous, well-designed, randomized, controlled trial. The quality of the endoscopic video recordings reflected methods that would not meet current standards for bowel preparation or recording procedures; however, the videos used in the current study were reassessed by blinded experienced central readers who were unaware of treatment strategy. Follow-up colonoscopies in the SONIC trial were only performed if an ulcer was identified at baseline. However, the presence of ulcers has become a common eligibility criterion in trials, making this a relevant sample to answer the study objectives. This study also did not examine the operating properties of endoscopic eligibility and outcomes employed in more recent phase 3 CD clinical trials. Although these trials subsequently led to regulatory approval of risankizumab and upadacitinib for the treatment of moderately to severely active CD, the endoscopic criteria employed have not yet been formally adopted by regulatory agencies and recommendations for endoscopic eligibility and outcome criteria in clinical trials may continue to evolve.

In conclusion, this study assessed the operating properties of several clinical and endoscopic indices commonly employed in CD registration trials and evaluated the sensitivity of various outcomes defined with these indices to detect a treatment effect when used alone and as coprimary or composite outcomes using a robust data set from a treatment of known efficacy. Although our findings generally corroborate current regulatory guidance and may assist regulatory agencies and investigators in the design of efficient clinical trials, these data will require validation in additional data sets, particularly those that include a placebo control, as well as data sets from trials involving therapies with differing mechanisms of action and therapeutic potential.

## Data Availability

The data underlying this article were generated based on video images and clinical data provided by Janssen. Data underlying this research may be shared on reasonable request to the corresponding author in consultation with Janssen.

## Abbreviations

ANOVA: analysis of variance
AP: abdominal pain
AZA: azathioprine
CD: Crohn’s disease
CDAI: Crohn’s Disease Activity Index
CDEIS: Crohn’s Disease Endoscopic Index of Severity
CRP: C-reactive protein
CI: confidence interval
FDA: Food and Drug Administration
ICC: intraclass correlation coefficient
IFX: infliximab
PRO: patient-reported outcome
SD: standard deviation
SES-CD: Simple Endoscopic Score for Crohn’s Disease
SF: stool frequency
SONIC: Study of Biologic and Immunomodulator Naive Patients in Crohn’s Disease
